# Correction: Zwitterionic pyrrolidene-phosphonates: inhibitors of the glycoside hydrolase-like phosphorylase *Streptomyces coelicolor* GlgEI-V279S

**DOI:** 10.1039/c7ob90121f

**Published:** 2017-08-09

**Authors:** Sri Kumar Veleti, Cecile Petit, Jared J. Lindenberger, Donald R. Ronning, Steven J. Sucheck

**Affiliations:** Department of Chemistry and Biochemistry and School of Green Chemistry and Engineering, The University of Toledo, 2801 W. Bancroft Street, Toledo, Ohio 43606, USA

The authors regret the omission of one of the authors, Jared J. Lindenberger, from the final version of the manuscript. The corrected list of authors and affiliations for this paper is as shown above.

The Royal Society of Chemistry apologises for these errors and any consequent inconvenience to authors and readers.

